# Cobalt and Iron Phthalocyanine Derivatives: Effect of Substituents on the Structure of Thin Films and Their Sensor Response to Nitric Oxide

**DOI:** 10.3390/bios13040484

**Published:** 2023-04-17

**Authors:** Darya Klyamer, Wenping Shao, Pavel Krasnov, Aleksandr Sukhikh, Svetlana Dorovskikh, Pavel Popovetskiy, Xianchun Li, Tamara Basova

**Affiliations:** 1 Nikolaev Institute of Inorganic Chemistry SB RAS, Novosibirsk 630090, Russia; klyamer@niic.nsc.ru (D.K.); a_sukhikh@niic.nsc.ru (A.S.); reter16@yandex.ru (S.D.); popovetskiy@niic.nsc.ru (P.P.); 2 School of Chemical Engineering, University of Science and Technology Liaoning, Anshan 114051, China; shaowp0318@163.com (W.S.); xianchunli@ustl.edu.cn (X.L.); 3 International Research Center of Spectroscopy and Quantum Chemistry, Siberian Federal University, Krasnoyarsk 660074, Russia; kpo1980@gmail.com

**Keywords:** iron phthalocyanines, cobalt phthalocyanines, singe crystals, thin films, chemi-resistive sensor, nitric oxide, quantum-chemical calculations

## Abstract

In this work, we study the effect of substituents in cobalt(II) and iron(II) phthalocyanines (CoPcR_4_ and FePcR_4_ with R = H, F, Cl, tBu) on the structural features of their films, and their chemi-resistive sensor response to a low concentration of nitric oxide. For the correct interpretation of diffractograms of phthalocyanine films, structures of CoPcCl_4_ and FePcCl_4_ single crystals were determined for the first time. Films were tested as active layers for the determination of low concentrations of NO (10–1000 ppb). It was found that the best sensor response to NO was observed for the films of chlorinated derivatives MPcCl_4_ (M = Co, Fe), while the lowest response was in the case of MPc(tBu)_4_ films. FePcCl_4_ films exhibited the maximal response to NO, with a calculated limit of detection (LOD) of 3 ppb; the response and recovery times determined at 30 ppb of NO were 30 s and 80 s, respectively. The LOD of a CoPcCl_4_ film was 7 ppb. However, iron phthalocyanine films had low stability and their sensitivity to NO decreased rapidly over time, while the response of cobalt phthalocyanine films remained stable for at least several months. In order to explain the obtained regularities, quantum chemical calculations of the binding parameters between NO and phthalocyanine molecules were carried out. It was shown that the binding of NO to the side atoms of phthalocyanines occurred through van der Waals forces, and the values of the binding energies were in direct correlation with the values of the sensor response to NO.

## 1. Introduction

Gas sensors play an important role in industry and agriculture in monitoring the composition of the surrounding atmosphere and determining the freshness of food. Another important area of their application is medical diagnostics. In a number of works, it has been shown that some diseases can be determined by the composition of exhaled air. For example, gases and vapors such as ammonia, nitric oxide, acetone and aldehydes can serve as important biomarkers of certain diseases [1]. For instance, an increased concentration of NO of more than 25 ppb in the exhaled air may indicate the presence of inflammatory processes in the respiratory tract [2,3,4].

The search for new materials for the creation of gas sensors with high sensitivity to low concentrations of analytes (up to the ppb level), good reproducibility, and selectivity is an urgent task of modern materials science. Semiconductor active layers, such as oxides, nitrides of transition metals, and carbon-containing materials, are widely used as sensor materials [5]. Despite a large number of studies, most modern semiconductor sensors have insufficient sensitivity to gaseous NO [6]. For this reason, the search for new materials with high sensitivity to gaseous NO is a very important task.

Metal phthalocyanines (MPc) are successfully used as sensing layers due to the possibility of varying their resistive, electrochemical and electrocatalytic properties across a wide range by changing the composition (e.g., substituents in macro-ring and central metal ions) or structural characteristics, and due to their high thermal and chemical stability compared to other organic compounds [7,8]. The advantages of MPc-based sensors also include their fast response times, good selectivity and the possibility of obtaining films on flexible substrates [9]. In comparison with sensors based on semiconductor oxides, MPc-based sensors demonstrate low time along with reversibility of the sensor response even at room temperature without additional heating.

Literature analysis shows that only a few papers describe the determination of nitric oxide in a gaseous medium using metal phthalocyanines and porphyrins [10,11,12,13,14]. However, a large number of works are devoted to the determination of NO metabolites (viz. NO_2_^2−^, NO_3_^2−^) in aqueous media by various electrochemical methods [15,16,17,18,19].

It is known that the sensor properties of metal phthalocyanines depend on their molecular structure. Previously, we studied the effect of the central metal, as well as the type of substituents and their position in the phthalocyanine ring, on the sensor response of MPc layers. For instance, Klyamer et al. [7,20] demonstrated that the central metals in MPcF_x_ (x = 4, 16) had a significant effect on the magnitude of the sensor response to ammonia, which decreased in the following series of metals: VO ~ Zn > Pb > Cu. It was also shown in our previous work [21] that the position and type of halogen (F or Cl) substituents affect the chemi-resistive sensor response to NH_3_. For instance, the sensor response of Cl-substituted zinc phthalocyanines was higher than that of their F-substituted analogues, and zinc phthalocyanines bearing halogen substituents in peripheral positions exhibited a higher value of response to NH_3_ compared to their analogues with substituents in non-peripheral positions. Knoben et al. [14] prepared monolayers of porphyrins 2H-PP, Co-PP, Fe-PP, and Zn-PP and studied their sensor characteristics. It was found that Zn-PP had the largest and fastest response to NO, and the ppb level of NO could be determined under ambient conditions. Nguyen et al. [22,23] used DFT calculation to study the interaction of nitric oxide (NO) with MPcs and various central metals, namely M = Mn, Fe, Co, Ni, Cu, Zn. It was shown that, among these phthalocyanines, FePc and CoPc had maximal energies for binding with NO. Similar to gaseous sensors, Ndebele and Nyokong showed in their work [19] that glassy carbon electrode modified with cobalt phthalocyanine derivatives displayed the best electrocatalytic activity for nitrite detection among other MPcs (M = Co, Cu, Mn, Ni). Considering these properties of cobalt and iron phthalocyanines, as well as the low time and the reversibility of the sensor response of phthalocyanine-based sensors at room temperature, these compounds were chosen as the subject of this study.

In this work, films of cobalt(II) and iron(II) phthalocyanines (CoPcR_4_ and FePcR_4_ with R = H, F, Cl, tBu) were studied as active layers of chemi-resistive sensors for the detection of low concentration of nitric oxide. The concentration of NO was analyzed in the range from 10 to 1000 ppb; however, the main attention was paid to determining the concentration of nitric oxide at the level of tens of ppb, since such concentrations are of interest for biological and medical applications. For example, in the clinical analysis of exhaled air, a NO level < 25 ppb is considered normal, 25–50 ppb is intermediate, and > 50 ppb is high [24,25]. The comparative analysis of films of phthalocyanines bearing various substituents allowed collection of the active layers with the best sensor sensitivity, detection limits, response and recovery times. MPcR_4_ films were deposited by thermal evaporation in a vacuum and their structure and morphology were investigated by X-ray diffraction (XRD) and microscopy methods. DFT calculations of the binding parameters between NO and CoPcR_4_ molecules were carried out to study the nature of the interaction and the regularities of the sensor response.

## 2. Materials and Methods

### 2.1. Synthesis of CoPcR_4_ and FePcR_4_ and Preparation of Their Films

**Figure 1:**
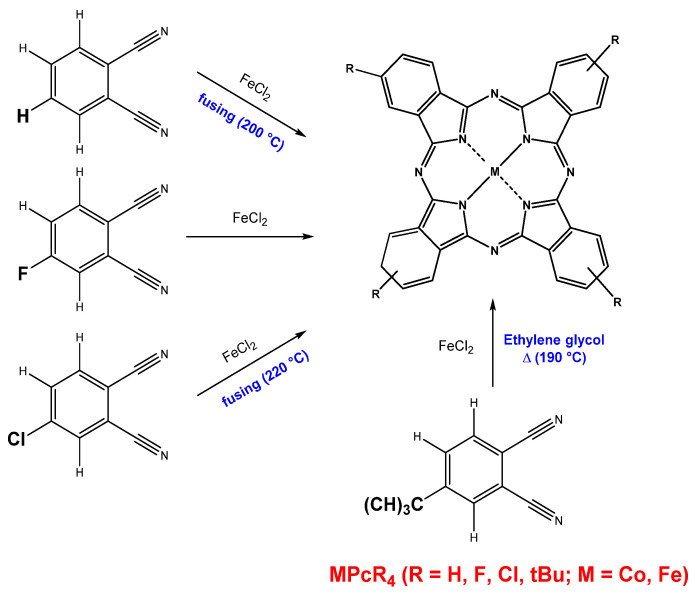
Synthetic route of MPcR_4_ (R = H, F, Cl, tBu; M = Co, Fe).

In this work, cobalt and iron phthalocyanine derivatives with four different substituents (Figure 1) were synthesized. Unsubstituted (MPc) and tetra-fluoro-substituted (MPcF_4_) iron and cobalt phthalocyanines were synthesized by the method of template synthesis (200 °C, 2 h) from metal chloride and phthalonitrile or 4-fluorophthalonitrile, respectively, according to the synthetic pathway described in the previous works [7,26].

CoPc: C_32_H_16_N_8_Co. Anal. Calc: C 67.3; H 2.8; N 19.6. Found: C 67.4; H 2.7; N 19.7. IR spectrum (KBr; ω, cm^−1^): 1609, 1591, 1522, 1468, 1425, 1333, 1288, 1165, 1121, 1088, 1001, 951, 912, 874, 779, 754, 571, 517, 434.

FePc: C_32_H_16_N_8_Fe. Anal. Calc: C 67.6; H 2.8; N 19.7. Found: C 67.2; H 2.8; N 19.6. IR spectrum (KBr; ω, cm^−1^): 1609, 1589, 1513, 1466, 1422, 1332, 1289, 1164, 1119, 1085, 1002, 949, 910, 804, 781 756, 573, 516, 436.

CoPcF_4_: C_32_H_12_F_4_N_8_Co. Anal. Calc: C 59.7; H 1.9; N 17.4, F 11.8. Found: C 60.1; H 2.0; N 17.5, F 12.0. IR spectrum (KBr; ω, cm^−1^): 1616, 1603, 1528, 1479, 1408, 1333, 1264, 1213, 1167, 1113, 1092, 1055, 955, 872, 820, 779, 750, 640, 515, 436.

FePcF_4_: C_32_H_12_F_4_N_8_Fe. Anal. Calc: C 60.0; H 1.9; N 17.5, F 11.9. Found: C 60.1; H 2.0; N 17.7, F 12.0. IR spectrum (KBr; ω, cm^−1^): 1616, 1600, 1518, 1485, 1407, 1334, 1264, 1167, 1113, 1087, 1053, 954, 945, 871, 750, 732, 642, 515, 436.

Tetra-chloro-substituted phthalocyanines (MPcCl_4_, M = Co, Fe) were synthesized by heating of a mixture of 4-chlorophthalonitrile (1 g, 4 mol) and iron(II) or cobalt(II) chloride (0.3 g, 1.5 mol) in a glass tube at 220 °C for 2 h. Then, the mixture was cooled to room temperature and crushed into powder.

CoPcCl_4_: C_32_H_12_Cl_4_N_8_Co. Anal. Calc: C 54.2; H 1.7; N 15.8. Found: C 54.4; H 1.7; N 15.9. IR spectrum (KBr; ω, cm^−1^): 1605, 1524, 1501, 1450, 1398, 1342, 1256, 1199, 1186, 1144, 1097, 1084, 1063, 966, 932, 883, 820, 775, 766, 750, 694, 636, 528, 430.

FePcCl_4_: C_32_H_12_Cl_4_N_8_Fe. Anal. Calc: C 54.4; H 1.7; N 15.9. Found: C 54.4; H 1.6; N 15.8. IR spectrum (KBr; ω, cm^−1^): 1605, 1516, 1456, 1396, 1329, 1308, 1256, 1202, 1185, 1142, 1076, 1045, 962, 928, 895, 885, 824, 775, 748, 694, 669, 624, 526, 432.

Tetra-tert-butyl phthalocyanines (MPc(tBu)_4_, M = Co, Fe) were synthesized according to the synthetic pathway previously described in [27]. The ground mixture of 4-tert-butylphthalonitrile (0.3 g, 4 mol) and iron(II) chloride (0.18 g, 1.5 mol) were refluxed in 3 mL of ethylene glycol at 190 °C for 5 h in argon atmosphere. Then, the mixture was cooled to room temperature and poured into water–ethanol solution (V_(ethanol)_:V _(water)_ = 1:1, 50 mL). The resulting precipitate was filtered and washed several times with the same ethanol–water mixture.

CoPc(tBu)_4_: C_48_H_48_N_8_Co. Anal. Calc: C 72.4; H 6.1; N 14.1. Found: C 72.2; H 6.1; N 14.2. IR spectrum (KBr; ω, cm^−1^): 1603, 1573, 1521, 1463, 1408, 1396, 1369, 1350, 1331, 1281, 1259, 1203, 1192, 1119, 1093, 1024, 927, 914, 895, 831, 756, 692, 663, 609, 536, 517, 434.

FePc(tBu)_4_: C_48_H_48_N_8_Fe. Anal. Calc: C 72.7; H 6.1; N 14.1. Found: C 72.7; H 6.0; N 14.2. IR spectrum (KBr; ω, cm^−1^): 1603, 1572, 1522, 1464, 1396, 1369, 1352, 1333, 1283, 1259, 1204, 1192, 1126, 1084, 1026, 928, 914, 897, 833, 750, 692, 663, 563, 517, 418.

The complexes obtained after synthesis were purified by double gradient sublimation in a (10^−5^ Torr, 400–450 °C). The structure of the MPcR_4_ phthalocyanines was verified using X-ray diffraction analysis, elemental analysis and FT-IR spectroscopy. Single crystals of MPcCl_4_ were obtained in the process of sublimation of the initial products.

Note that, in the case of tetrasubstituted complexes of metal phthalocyanines, substituents can be introduced into both peripheral and non-peripheral positions of the phthalocyanine ring. In this paper, only phthalocyanine derivatives with substituents in the peripheral positions of the phthalocyanine ring were considered, due to the fact that they have a significantly higher sensor response toward electron donor gases compared to non-peripherally substituted examples [21,28].

Thin films were obtained by organic molecular beam deposition on glass substrates or glass slides with pre-deposited Pt interdigitated electrodes (IDE). The substrate temperature was about 60 °C. The nominal thickness of the films was about 75–90 nm. The films’ thickness was controlled by a quartz crystal microbalance and verified by spectral ellipsometry, as described in our previous work [29].

### 2.2. Characterization of Metal Phthalocyanines and Their Films

Structures of single crystal of MPcR_4_ were determined using a single-crystal diffractometer Bruker D8 VENTURE with the following characteristics: MoKα λ = 0.71073 Å Incoatec IμS 3.0 microfocus source, PHOTON III C14 CPAD detector, 3-circle goniometer). The diffractometer was equipped with an open-flow nitrogen cooler (Oxford cryo-systems Cryostream 800 plus), which allowed maintenance of a sample temperature at 150(1)K. To collect data, several standard ω scans were performed with frames 0.5° wide. APEX3 V2018.7-2 (SAINT 8.38A, SADABS-2016/2) was used for data collection and reduction, unit cell refinement and absorption correction [30]. In order to solve and refine structure, F_hkl_ datasets were processed in Olex2 v.1.5 [31] using SHELXT 2018/2 [32] and SHELXL 2018/3 [33]. Diffraction patterns of MPcR_4_ films and powders were obtained using a Bruker D8 Advance powder diffractometer (Cu-anode sealed tube, 40 mA @ 40 kV, LYNXEYE XE-T compound silicon strip detector) in the Bragg-Brentano geometry. The scan step was 0.01023° and the acquisition time was 2 s/step.

Atomic force microscopy (AFM) images of MPcR_4_ films were obtained using a Ntegra Prima II (NT-MDT, Russia) microscope in semi-contact mode. The HA_NC tip with Au reflective side (TipsNano, Estonia) had a length of 124 μm, a width of 34 μm and a thickness of 1.85 μm. The force constant was 3.5 N/m, while the resonance frequency was 140 kHz. Nova SPM software was utilized to calculate roughness parameters according to the standards ISO 4287-1, ISO 4287 and ASME B46. Scanning electron microscopy (SEM) images were obtained on a scanning electron microscopes JEOL 6700F.

### 2.3. Study of the Sensor Properties of MPc Films

To test the chemi-resistive sensor response, films were deposited onto commercial platinum IDE (Dropsens, Spain). The IDE parameters were as follows: the number of digits was 125 × 2, while the gap between digits was 10 μm. Phthalocyanine films deposited onto IDE were placed in a gas flow cell and kept for 30 min in an argon stream until their resistance reached a steady value. Argon was chosen as carrier and diluent gas due to the fact that nitric oxide (NO) is highly reactive and easily oxidizes to nitrogen dioxide (NO_2_) in air. The required gas flow was regulated using mass flow regulators. The resistance of MPcR_4_ films was measured using a Keithley 236 electrometer (constant DC voltage = 10 V). Injection of NO was carried out at the constant air flow rate of 1000 mL/min and the exposure time was fixed at 15 s.

The sensor response was investigated in dynamic mode with constant argon purging. Static mode was used to measure response and recovery times. In this mode, the cell was first purged with argon, then, when a constant resistance value was reached, the argon supply was turned off and a mixture of gases containing the required concentration of NO was introduced into the cell. After saturation of the sensor layer, argon purging was resumed.

### 2.4. Quantum-Chemical Calculations

**Figure 2:**
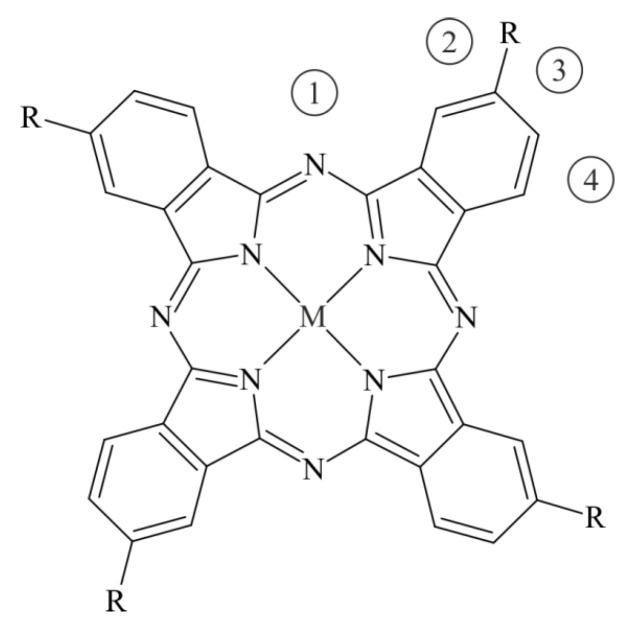
The structural formula of MPcR_4_ monomeric phthalocyanines with C_4h_ symmetry. Numbers in circles denote nonequivalent attachment sites of the NO molecule.

Quantum-chemical calculations of the interaction of the NO molecule with phthalocyanines in the form of MPcR_4_ monomers and 2MPcR_4_ dimers (M = Co, Fe; X = H, F, and Cl) were performed with the help of the ORCA software package [34,35], using the DFT BP86-D3/def2-SVP method [36,37,38,39,40], RI approximation [41,42,43,44,45,46], and the corresponding auxiliary basis set Def2/J [47]. The introduction of four substituents (one in each benzene ring) leads to the formation of four isomers that differ in the mutual arrangement of substituents, which, in the case of a planar macrocycle, corresponds to the D_2h_, C_2v_, C_4h_, and C_s_ point symmetry groups of the substituted molecule. In this situation, the molecule has several nonequivalent adsorption centers for gas molecules that bind through the side atoms of the macrocycle. At the same time, considering separately the interaction of an NH_3_ molecule with CoPcF_4_ and VOPcF_4_ through four bridge nitrogen atoms, we showed that the parameters of this interaction were very close in all four cases [28]. In this regard, in order to simplify calculations by reducing the number of possible nonequivalent adsorption centers for the NO molecule, MPcR_4_ molecules with C_4h_ symmetry (Figure 2) were considered in this work, although symmetry constraints were not used in the calculation process. In this case, four different places were identified for the formation of a bond between nitric oxide and side atoms of macrocycles—three around the benzene ring and one opposite the bridge nitrogen atom. Further, aggregates of monomeric phthalocyanines with the NO molecule will be designated as MPcR_4_/NO-m, where m is 1–4 depending on the site of adsorption of NO. Note that, in the case of unsubstituted MPcs, the aggregates MPc/NO-2 and MPc/NO-4 are equivalent, so only the first of them will be considered. Aggregates of dimeric phthalocyanines will be designated as 2MPcR_4_/NO.

Since the considered compounds have an open electron shell, the calculations were performed according to the spin-unrestricted Kohn-Sham (UKS) theory. In this case, the spin multiplicity was equal to one for 2CoPcR_4_ dimers, two for NO molecule, CoPcR_4_ monomers, and 2CoPcR_4_/NO aggregates, three for FePcR_4_ monomers and CoPcR_4_/NO-m aggregates, four for FePcR_4_/NO-m aggregates, five for FePcR_4_ dimers, and six for 2FePcR_4_/NO aggregates. Preliminary calculations have shown that these spin states were energetically more favorable.

In the process of calculation, the geometry of all considered compounds was first optimized, followed by the calculation of their vibrational spectra, to make sure that there were no imaginary frequencies, since this, along with the minimum of the total energy, is the criterion for achieving an equilibrium state. Then the binding energy *E_b_* of the NO molecule with monomeric or dimeric phthalocyanines was calculated from the difference in the total energies of both aggregate parts and the aggregate itself

(1) $$E_{b}=E_{\mathrm{NO}}+E_{{(2)\mathrm{MPcR}}_{4}}-E_{{(2)\mathrm{MPcR}}_{4}/\mathrm{NO}}-\Delta E_{\mathrm{BSSE}}$$

where Δ*E*_BSSE_ is the correction to the binding energy, taking into account the basis set superposition error (BSSE), which is estimated as follows:

(2) $$\Delta E_{\mathrm{BSSE}}=\left(E_{{(2)\mathrm{MPcR}}_{4}}^{*}+E_{\mathrm{NO}}^{*}\right)-\left(E_{{(2)\mathrm{MPcR}}_{4}*}^{*}+E_{\mathrm{NO}*}^{*}\right)$$

Here, the asterisk in the superscript means that the corresponding total energies were calculated for MPcR_4_ (2MPcR_4_) and NO compounds separated from the equilibrium structure of the aggregate, without subsequent optimization of their geometric structure. An asterisk in the lower index indicates that, instead of the atoms of the second fragment of the entire aggregate, points described by the corresponding sets of atomic orbitals were considered.

The next step was a topological analysis of the electron density ρ(**r**) distribution in the MPcR_4_/NO-m and 2MPcR_4_/NO aggregates, performed in the framework of the QTAIM theory [48,49,50] using the AIMAll software package [51]. For this purpose, the electronic wave functions of these structures were calculated using a similar method and the cc-pVTZ basis set of atomic orbitals [52] instead of def2-SVP for greater accuracy. As a result, the values of the electron density, its Laplacian ∇^2^ρ(**r**), and the electronic energy density h(**r**) at bond critical points (BCPs) characterizing the interaction of the NO molecule with the side atoms of phthalocyanines were obtained.

## 3. Results and Discussion

### 3.1. Single Crystal Structure of CoPcCl_4_ and FePcCl_4_

In order to correctly interpret XRD patterns of phthalocyanine films, it is necessary to have data on the structure of their single crystals. The structures of CoPc, FePc, CoPcF_4_, and FePcF_4_ single crystals have already been determined in previous works [53,54,55,56]. Single crystals of CoPcCl_4_ and FePcCl_4_ were grown by sublimation in a vacuum and their structures were determined for the first time.

**Figure 3:**
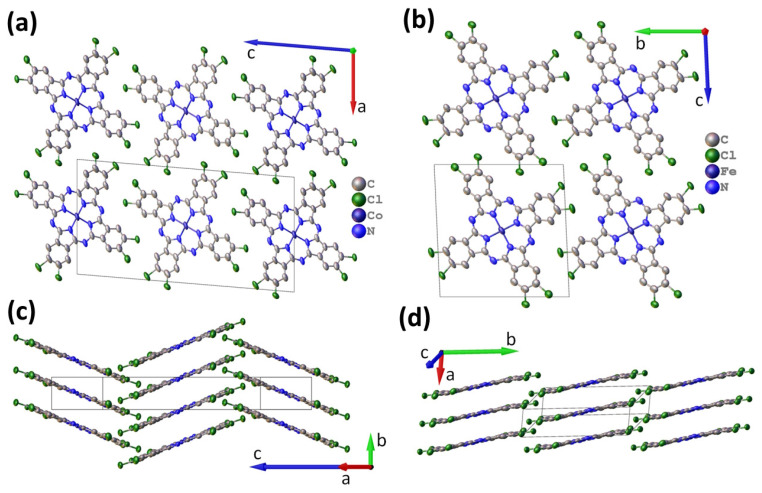
Molecular packing diagrams for CoPcCl_4_ (**a**,**b**) and FePcCl_4_ (**c**,**d**).

CoPcCl_4_ crystallizes in a P2_1_/c space group with Z = 2 and is isostructural to the previously reported CuPcCl_4_ [57]. CoPcCl_4_ molecules are packed into stacks (Figure 3a), which form a “herringbone” pattern when viewed from the side (Figure 3b). The detailed refinement statistics and unit cell parameters are given in Table 1. CoPcCl_4_ molecules are relatively flat; the maximum deviation from the mean squared plane is less than 0.1 Å for any atom in the molecule (except hydrogen). The packing angle (angle between the line through the central metal atoms and the normal to the least-squares plane, through all atoms in the phthalocyanine molecule except hydrogen) is 21.17° for CoPcCl_4_. The angle between molecules in adjacent stacks is 42.34°. The distance between neighboring molecules in the stack is 3.376 Å, while the distance between neighboring Co atoms is 3.620 Å. For comparison, the packing angle in CuPcCl_4_ is 21.67°, while the distance between neighboring molecules is 3.381 Å and the distance between Cu atoms is 3.638 Å. For unsubstituted β-CoPc [58], the distance between molecules in the stack is 3.320 Å, the stacking angle is 45.93°, and the distance between neighboring Co atoms is 4.773 Å, while for α-CoPc [55] and tetra-fluorinated CoPcF_4_ [54], these values are 3.425 Å/24.16°/3.754 Å and 3.322 Å/24.58°/3.653 Å, respectively. In general, if the CoPcCl_4_ packaging is similar to the “herringbone” packing motif of β-CoPc, then the arrangement of CoPcCl_4_ molecules within the stack is more is more like the arrangement in α-CoPc and CoPcF_4_.

Since a mixture of four regio-isomers is formed during the synthesis of MPcCl_4_-p, the CoPcCl_4_ structure contains two symmetrically independent chlorine atoms, each of which is disordered over two positions, with a total occupancy equal to 1. In CoPcCl_4_, the chlorine atom occupancy ratios are 0.514(3)/0.486(3) and 0.531(3)/0.469(3). The fact that these ratios are close to 0.5/0.5 indicates that there are no preferred positions for Cl atoms.

FePcCl_4_ crystallizes in P-1 space group with Z = 1 and is isostructural to FePcF_4_ [53], as well as to α-polymorphs of unsubstituted MPcs, e.g., α-CuPc [59] (Table 1). FePcCl_4_ molecules are packed into uniform stacks, with molecules in adjacent stacks parallel to each other (Figure 3). FePcCl_4_ molecules are less flat than CoPcCl_4_, with the maximum deviation from mean squared plane equal to 0.125 Å. The packing angle for FePcCl_4_ molecules is 19.63°, the distance between adjacent molecules is 3.388Å and the distance between neighboring Fe atoms is 3.597 Å. For comparison, the packing angle in FePcF_4_ is 24.06°, the distance between molecules is 3.332 Å and the distance between Fe atoms in neighboring molecules is 3.649 Å, while for unsubstituted β-FePc [60] these values are 3.301 Å/46.33°/4.781 Å. The FePcCl_4_ molecule contains two symmetrically independent chlorine atoms, each of which is disordered over two positions with occupancy ratios of 0.484(3)/0.516(3) and 0.443(3)/0.557(3).

**Figure 4:**
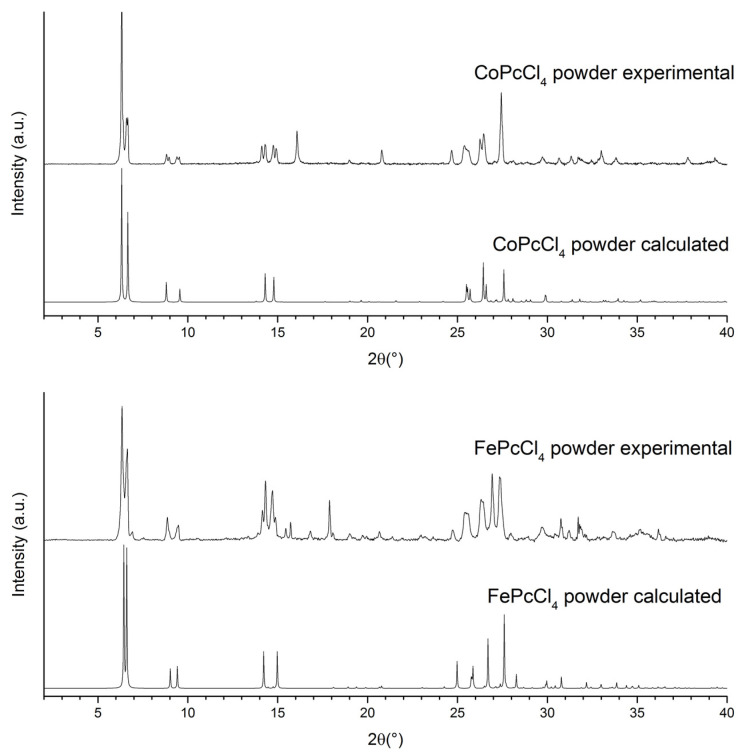
Experimental and calculated powder diffraction patterns of CoPcCl_4_ and FePcCl_4_ (CuKα, λ = 1.54187 Å).

Figure 4 shows experimental powder diffraction patterns of CoPcCl_4_ and FePcCl_4_ in the 2θ range of 2–40° in comparison with those calculated from their single-crystal data. It is clear that both experimental diffraction patterns do not completely coincide with the corresponding calculated patterns and contain one additional crystal phase in a comparable quantity (this is especially noticeable in the range from 8° to 15° of the diffraction patterns in Figure 4). For example, the calculated diffraction pattern of CoPcCl_4_ contains only four diffraction peaks in the 2θ range of 5–10° (6.31°, 6.67°, 8.80°, 9.56°), while the experimental diffraction pattern shows four additional diffraction peaks in the same region (6.41°, 6.60°, 8.96°, 9.40°), which cannot be explained using the CoPcCl_4_ single-crystal data.

Despite all our efforts, we were unable to find single crystals for the second phase for either CoPcCl_4_ or FePcCl_4_. However, it should be noted that additional four diffraction peaks in the region of 5–10° on the CoPcCl_4_ diffraction pattern coincide very well with the first four peaks calculated from the FePcCl_4_ single-crystal data (6.43°, 6.60°, 9.00°, 9.42°). The same, but in reverse order, is true for the experimental powder pattern of FePcCl_4_. This is a very strong argument in favor of the fact that the second crystal phase of CoPcCl_4_ is isostructural with the first crystal phase of FePcCl_4_ and vice versa, and that the bulk powders of CoPcCl_4_ and FePcCl_4_ contain both monoclinic and triclinic polymorphs.

### 3.2. Thin Films of CoPcR_4_ and FePcR_4_ (R = H, F, Cl, tBu)

The films of CoPcR_4_ and FePcR_4_ were deposited by a PVD method. The composition of films coincides with that of powders, which is confirmed by Raman spectroscopy. The Raman spectra of thin films and powders of CoPcCl_4_ and FePcCl_4_ are shown in Figure S1 (Supporting Information). Raman spectra of other investigated phthalocyanines were studied in previous works [26,53].

**Figure 5:**
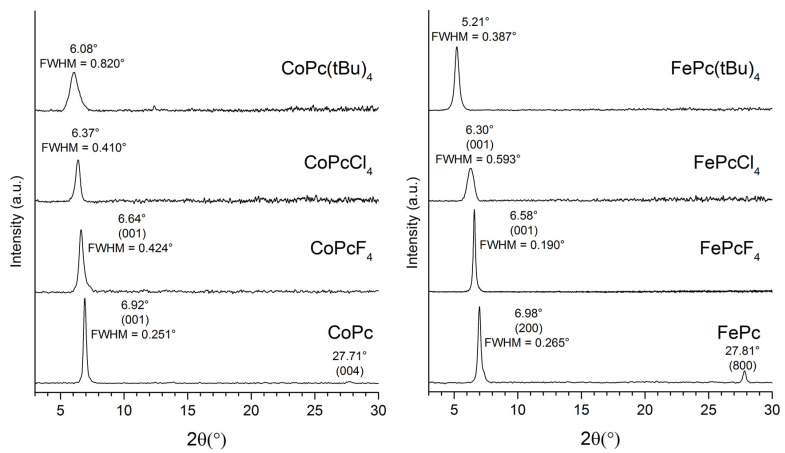
XRD patterns of CoPcR_4_ and FePcR_4_ (R = H, F, Cl, tBu) films.

XRD patterns of CoPcR_4_ and FePcR_4_ thin films (R = H, F, Cl, tBu) are shown in Figure 5. One strong diffraction peak is observed in all diffraction patterns, which indicates a strong preferred orientation of phthalocyanine crystallites relative to the substrate surface in these films. A strong peak at 6.92° on the XRD pattern of CoPc film coincides well with the (001) peak of α-CoPc at 6.91° [55]. An additional weak diffraction peak is visible at 27.71°, which corresponds to the (004) plane. A strong peak at 6.98° on the XRD pattern of FePc can be attributed to the (200) peak of α-FePc at 6.94° [56], while a diffraction peak at 27.83° corresponds to the (800) peak of α-FePc. The XRD patterns of both CoPcF_4_ and FePcF_4_ films have single strong diffraction peaks, which correspond to the (001) plane of the respective crystal phases (6.62° for CoPcF_4_ and 6.64° for FePcF_4_, as calculated from single-crystal data) [53,54].

The relatively wide diffraction peak on the CoPcCl_4_ thin film XRD pattern may coincide with the first peak of either the monoclinic or triclinic phase. The same is partially true for the FePcCl_4_ thin film; however, judging by the position of the peak, it most likely refers to the (100) peak of the monoclinic phase. Finally, XRD patterns of CoPc(tBu)_4_ and FePc(tBu)_4_ films also have one wide diffraction peak. Since there is no data for CoPc(tBu)_4_ and FePc(tBu)_4_ single crystals, and tert-butyl substituted phthalocyanines usually tend to be amorphous, we cannot say whether CoPc(tBu)_4_ and FePc(tBu)_4_ films have a preferred orientation or not. The only conclusion, judging by the difference in the positions of the peaks on the corresponding XRD patterns, is that CoPc(tBu)_4_ and FePc(tBu)_4_ films have completely different styles of molecular packaging.

Knowing the FWHM values of the observed diffraction peaks, the coherent scattering region size for each thin film can be estimated using the Scherrer equation. Taking into account the instrumental peak broadening of 0.05° (measured using SRM-660a LaB_6_ powder), the following values were obtained: 44 nm for CoPc, 24 nm for CoPcF_4_-p, 25 nm for CoPcCl_4_-p, and 11.5 nm for CoPc(tBu)_4_. The values for FePcR_4_ films were 41, 63, 16.3, and 26 nm, respectively.

**Figure 6:**
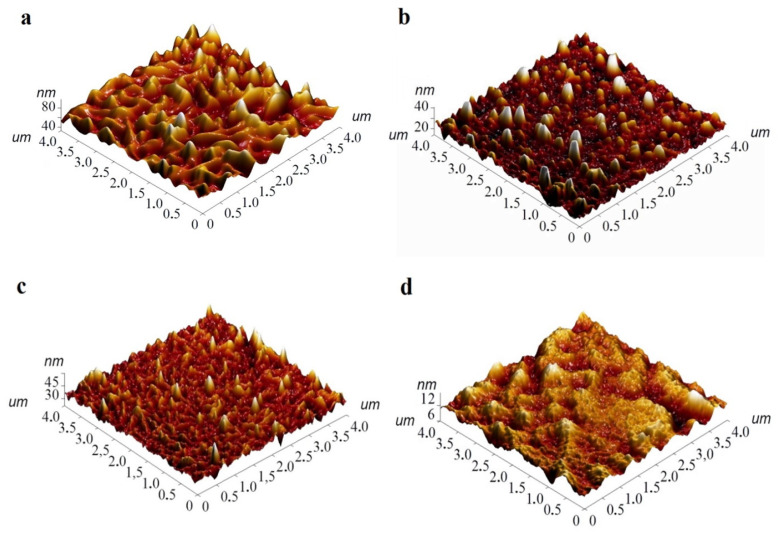
3D AFM images of FePc (**a**), FePcF_4_ (**b**), FePcCl_4_ (**c**), and FePc(tBu)_4_ (**d**) films.

The morphology of the films was studied by AFM. Figure 6 shows 3D AFM images for FePcR_4_ films as an example. 2D AFM images are given in the Supporting Information (Figure S2). SEM images are also presented in Figure S3. All investigated films have different surface morphology. For example, the surface of a FePc film consists of elongated crystallites, with length reaching 0.8 μm. The root mean square (RMS) roughness value of a FePc film is 9.5 nm. The film of FePcF_4_ consists of the clearly distinguishable smaller roundish grains combined in bigger aggregates and has RMS roughness of 5.0 nm. The morphology of the FePcCl_4_ film differs significantly from that of the films of FePcF_4_; the film is formed by thin elongated crystallites, the size of which reaches 0.5 μm. Its RMS roughness value is 4.5 nm. The film of FePc(tBu)_4_ has no clearly visible crystallites, consists of big aggregates, and its RMS roughness value is 3.8 nm.

### 3.3. Sensor Properties of CoPcR_4_ and FePcR_4_ (R = H, F, Cl, tBu) Films

**Figure 7:**
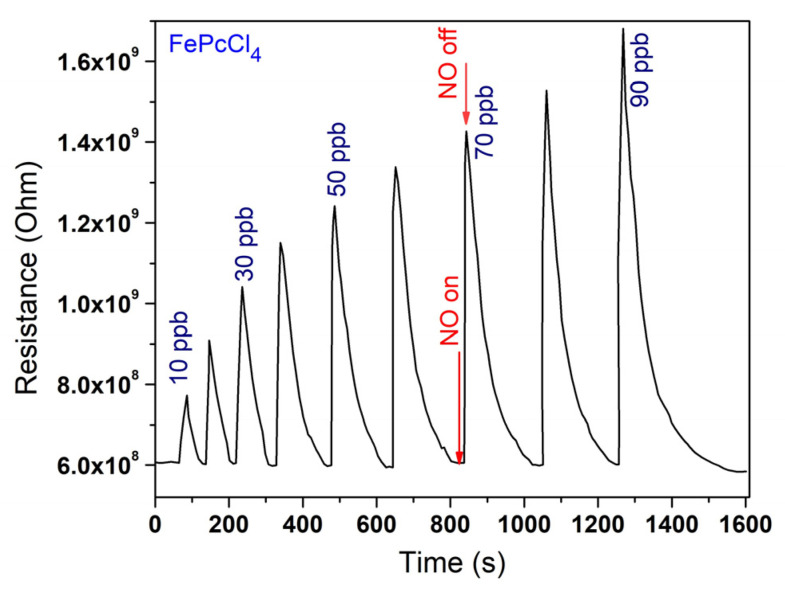
Typical sensor response of a FePcCl_4_ film to NO (10–90 ppb).

The films of CoPcR_4_ and FePcR_4_ films were deposited on glass substrates with IDE Pt electrodes for the investigation of the sensor response to nitric oxide. Figure 7 shows a typical sensor response of FePcCl_4_ film as an example. Similarly to the case of the sensor response of MPcF_4_ films to ammonia [28], when NO was introduced into the cell, a sharp increase in resistance was observed, and after purging with argon, the resistance returned to its original value. With an increase in NO concentration, the change in resistance increased.

**Figure 8:**
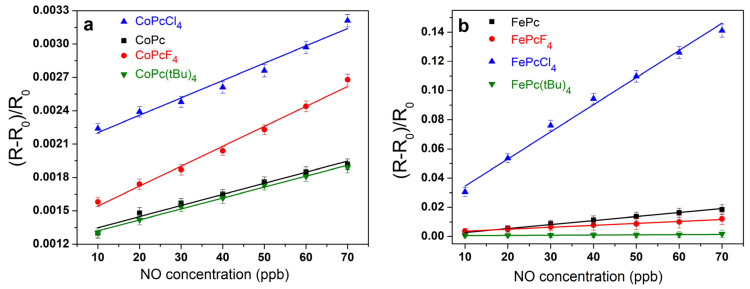
Dependence of the sensor response of CoPcR_4_ (**a**) and FePcR_4_ (**b**) (R = H, F, Cl, tBu) films on NO concentration (10–70 ppm).

The sensor response was defined as S_resp_ = (R − R_0_)/R_0_, where R_0_ is the initial resistance of the film in argon atmosphere and R is the resistance of the phthalocyanine film at a certain NO concentration. The influence of different types of substituents in MPcR_4_ on the chemi-resistive response to nitric oxide (10–70 ppb) was studied to select the material with the best sensor characteristics. The dependences of the sensor response on NO concentration are shown in Figure 8 for all investigated MPcR_4_ films.

Comparison of the sensor response of iron and cobalt phthalocyanine films with various substituents showed that the best sensor response was observed for chlorinated derivatives MPcCl_4_ (M = Co, Fe), while the lowest response was for MPc(tBu)_4_ films. For example, the sensor response of a CoPcF_4_ film to 30 ppb NO was 1.2 times higher than that of a CoPc film, while the response of CoPcCl_4_ to NO was 1.6 times higher than that (Figure 8a). In the case of iron phthalocyanines derivatives, sensor response of a FePcCl_4_ film to 30 ppb of ammonia was more than 11 times higher than for FePcF_4_ and FePc films and about 90 higher than in the case of Fe(tBu)_4_ (Figure 8b).

**Figure 9:**
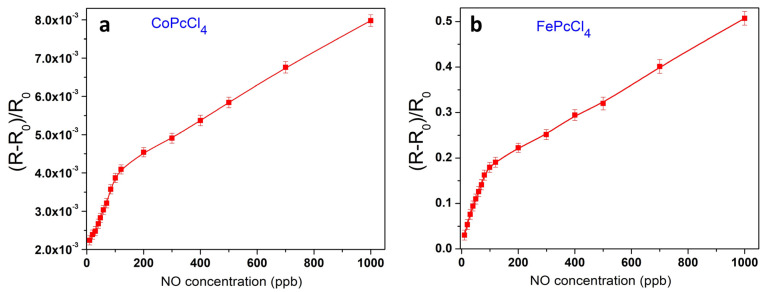
Dependence of the sensor response of CoPcCl_4_ (**a**) and FePcCl_4_ (**b**) films on NO concentration (10–1000 ppb).

The dependence of the sensor response of CoPcCl_4_ and FePcCl_4_ films, which have the best sensor characteristics, on NO concentration was studied in a wider concentration range from 10 to 1000 ppb (Figure 9). The curves have two linear ranges: from 10 to 90 ppb and from 100 to 1000 ppb. In the whole range, a reversible sensor response was observed.

The average sensor responses to 30 ppb of nitric oxide, as well as the response and recovery time of all investigated films, are summarized in Table 2.

**Figure 10:**
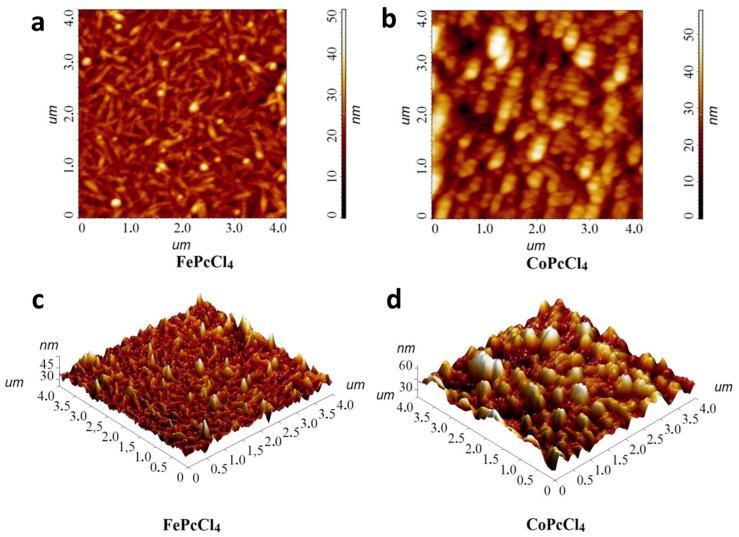
2D and 3D AFM images of FePcCl_4_ (**a**,**c**) and CoPcCl_4_ films (**b**,**d**).

The calculated limits of detection (LOD) of NO, defined as 3σ/m, where σ is the standard deviation of the sensor response to 10 ppb NO and m is the slope of the corresponding calibration plot (Figure 8) in the linear region (10–90 ppb), are also given in Table 2. The response of iron phthalocyanine films, with the exception of FePc(tBu)_4_, is several times higher than the response of cobalt phthalocyanine films. Among the investigated sensors, FePcCl_4_ film exhibited the maximal response to NO, with response and recovery times (determined at 30 ppb of NO) of 30 s and 80 s, respectively. Its calculated detection limit was 3 ppb. At the same time, the LOD of a CoPcCl_4_ film was 7 ppb. In addition to the molecular structure of the complex, the sensor response is influenced by the morphology and structural features of thin films (Figure 10). CoPcCl_4_ films are formed by rounded crystals, the size of which reaches 100 nm. The morphology of the FePcCl_4_ film differs significantly from the morphology of FePcF_4_ films; the film is formed by thin elongated crystallites, the size of which reaches 0.6 μm.

**Figure 11:**
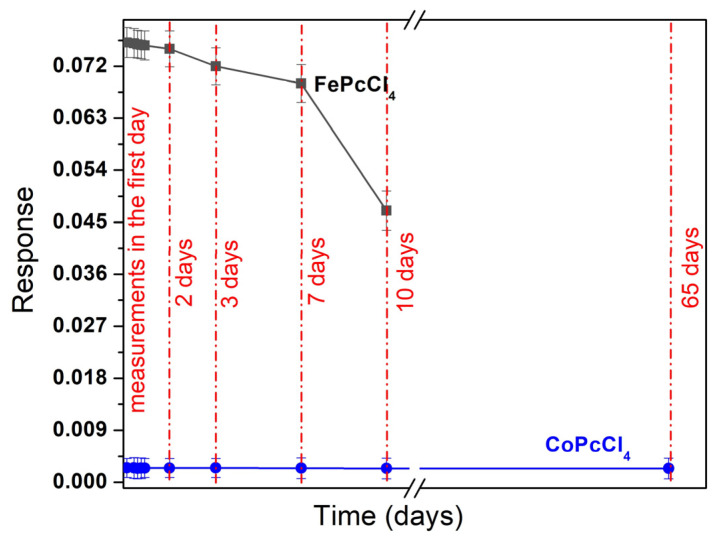
Change of the sensor response of FePcCl_4_ and CoPcCl_4_ films to NO (30 ppb) over time.

Further tests of the repeatability and stability of the films showed that iron phthalocyanine films had low stability and their sensor response dropped rapidly. The sensor response of a fresh FePcCl_4_ film to 30 ppb of nitric oxide and the same film after 2, 3, 7, and 10 days is shown in Figure 11. The change in the sensor response within 2 days did not exceed the measurement error, but after 7 days the response decreased dramatically. At the same time, all cobalt phthalocyanine films remained stable, at least for several months.

**Figure 12:**
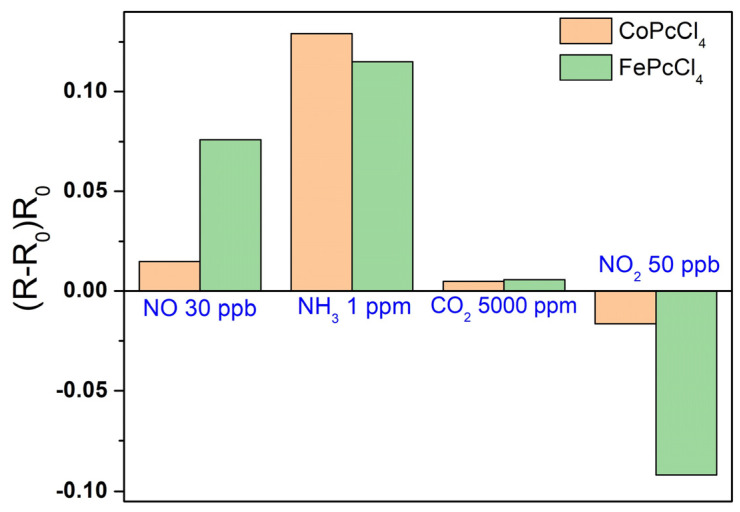
Sensor response of FePcCl_4_ and CoPcCl_4_ films to NO (30 ppb), NH_3_ (3 ppm), and CO_2_ (5000 ppm) and NO_2_ (50 ppb).

The sensor response of FePcCl_4_ and CoPcCl_4_ films to NO was also compared with that to ammonia (NH_3_), carbon dioxide (CO_2_) and nitrogen dioxide (NO_2_) (Figure 12), which shows that small concentrations of NO (at ppb level) can be detected in the presence of CO_2_, but ammonia at concentrations of the ppm level can interfere with the determination of nitric oxide.

One more interfering gas is nitrogen dioxide (NO_2_), which is a strong electron acceptor. In contrast to the sensor response to NO, the introduction of NO_2_ to the flow cell leads to a decrease in the resistance of FePcCl_4_ and CoPcCl_4_ layers. It is also necessary to mention that determination of NO can be performed only in a strictly controlled inert atmosphere due to its low stability. One of the approaches for determining NO in air is the quantitative oxidation of NO to NO_2_, followed by a study of the chemi-resistive sensor response to NO_2_ [61].

Thus, active layers based on chloro-substituted phthalocyanines of cobalt and iron have a sufficiently high sensitivity to NO, fast reversible response and low recovery time at room temperature, and their characteristics are comparable, or in some parameters even exceed, the characteristics of sensors based on other materials [62,63,64,65]. Most chemi-resistive sensors based on semiconductor oxides operate only at elevated temperatures. For example, sensors based on iron oxide nanorods exhibited reversible sensor response to NO in the concentration range from 0.5 ppm to 2.75 ppm at 250 °C [62]. Su and Li [63] reported a chemi-resistive gas sensor made of composite films with the complex structure Fe_2_O_3_/MWCNTs/WO_3_ modified with noble metals, which demonstrated a reversible sensor response to NO at room temperature, but the minimal detected concentration of NO was 100 ppb.

### 3.4. Quantum-Chemical Modeling of the Interaction between NO and MPcR_4_ Molecules

The most common accepted interpretation of the mechanism of chemi-resistive sensor response to NO is that the effect of gaseous NO on metal phthalocyanine films, which usually behave like p-type semiconductors, leads to a change in their conductivity due to depletion of positively charged holes by electrons donated by the NO [22]. At the same time, the place of the adsorption of gas molecules on the surface of phthalocyanine films remains a subject of discussion.

To study the nature of interaction between NO and phthalocyanine molecules and to explain the effect of R in MPcR_4_ phthalocyanines on the sensor response of their films to nitric oxide, quantum-chemical modeling of the NO molecule interaction with MPcR_4_ (R = H, F, Cl) molecules was carried out. It is necessary to mention that the calculation for MPc(tBu)_4_ was not performed because, firstly, the sensor response is much less than that of MPcR_4_ with R = H, Cl, F. Secondly, molecules in films of MPcR_4_ (R = H, F, Cl) are packed in stacks, and their dimers, considered in the process of quantum chemical calculations, are fragments of these stacks. The structure of MPc(tBu)_4_ films differs from others and, as XRD analysis shows, the films are less crystalline and possibly disordered due to the steric effects of tert-butyl groups. This does not allow us to consider the same calculation models for them as for MPcR_4_ (R = H, F, Cl) films.

**Figure 13:**
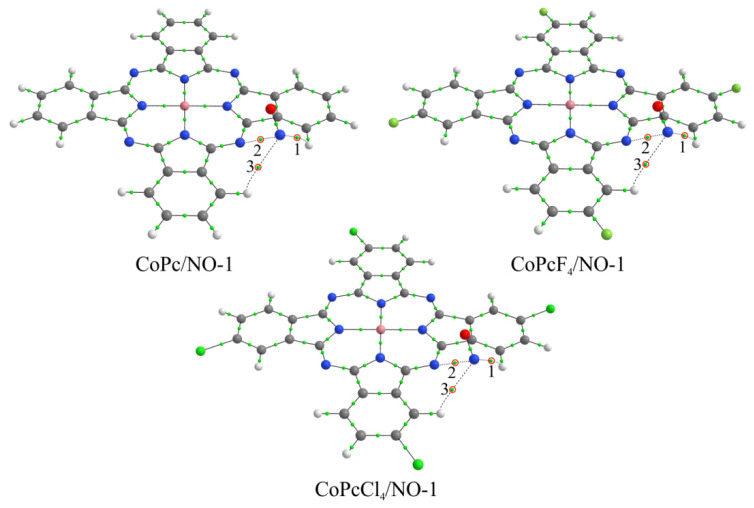
Structure of CoPcR_4_/NO-1 aggregates, where R = H, F, and Cl, along with bond critical points. Red circles and numbers 1–3 indicate BCPs characterizing the NO molecule interaction with phthalocyanine atoms.

**Figure 14:**
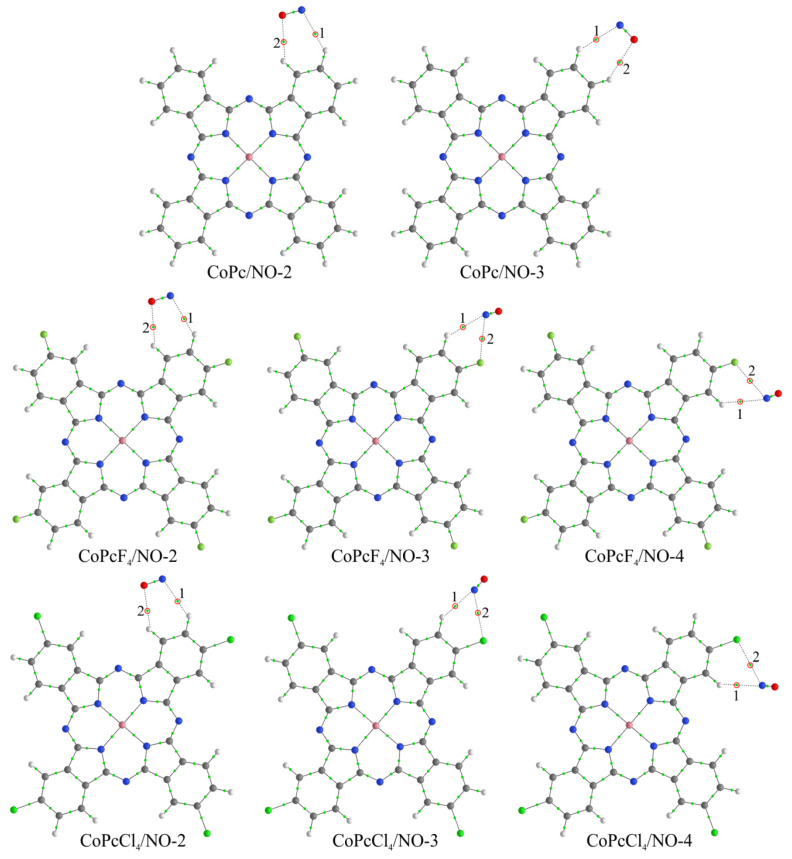
Structure of CoPcR_4_/NO-*m* aggregates, where R = H, F, and Cl, m = 2–4, and bond critical points in them. Red circles and numbers 1 and 2 indicate BCPs characterizing the NO molecule interaction with phthalocyanine atoms.

Despite the fact that strong binding should be observed in the case of interaction with a central metal atom [22], when the binding energies reach 1.5 eV for CoPc and 1.9 eV for FePc, depending on the orientation of the nitric oxide molecule, we did not consider this method of coordination for two reasons [28]. First, due to the *π*-*π* interaction, the typical distance between two phthalocyanine molecules in a stack is too small for any gas molecule to penetrate between them. This distance is about 3.4 Å [66], which roughly corresponds to the van der Waals diameter of a carbon atom. Second, the indicated values of binding energy of the NO molecule with metal atoms are large enough for the desorption of nitric oxide under normal conditions [21]. Therefore, the sensor response must be irreversible, which contradicts the experimental data presented above. Moreover, Chia et al. [67] demonstrated by in-situ X-ray absorption spectroscopy (XAS) and EXAFS that NO_2_ interacts with CuPc at the pyrrole moiety of the Pc macrocycle, rather than on the metal center. In this regard, the simulated interaction was carried out through the side atoms of the macrocycles (Figure 13 and Figure 14), similar to previous studies [21,28]. 

As a result, it was found that, in the case of monomeric phthalocyanines, the most energetically favorable method of NO molecule binding is its interaction through the bridge nitrogen atom (MPcR_4_/NO-1 aggregates) (Figure 13), when the *E_b_* values are higher (Table 3). However, in these compounds, nitric oxide is actually located above the plane of the macrocycle, and in the case of a stack, this would mean that the gas molecule is located between two phthalocyanine molecules. However, as has already been mentioned above, the distance between two molecules in the stack is not enough for the location of NO molecules. For this reason, this aggregate is not of interest for further consideration.

The binding energy of nitric oxide at the other three positions (two in the case of MPc/NO) is very low (Table 3). This indicates that these methods of NO molecule coordination cannot have a significant effect on the change in the electrical conductivity of phthalocyanine films, and therefore are also of no interest for further consideration. In general, the low binding energies between NO and phthalocyanine molecule with phthalocyanines in MPcR_4_/NO-*m* aggregates are evidence of the van der Waals interaction between them. This is supported by the results of a topological analysis of the electron density distribution in these compounds, which made it possible to establish the corresponding BCPs between the atoms of nitrogen oxide and phthalocyanines (Figure 13 and Figure 14). In particular, it is shown that the values of ∇^2^*ρ*(**r**) and *h*(**r**) at these points are positive (Table S1, Supplementary Materials).

**Figure 15:**
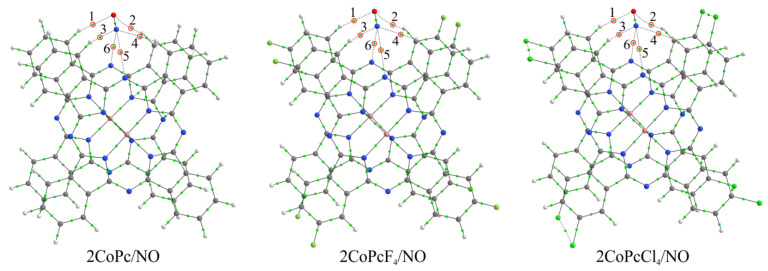
Structure of 2CoPcR_4_/NO aggregates, where R = H, F, and Cl, and bond critical points in them. Red circles and numbers 1–6 indicate BCPs characterizing the NO molecule interaction with phthalocyanine atoms.

This means that the so-called closed-shell interaction is observed here, which is characteristic of ionic, highly polar covalent, hydrogen, and van der Waals bonds [48,49,50]. However, low values of *ρ*(**r**), in most cases less than 0.01 a.u., testify in favor of the latter. Previously, it was shown that, for van der Waals bonds, the character values of the electron density are of the order of 10^−3^ a.u. [48,68]. The exception here is the MPcR_4_/NO-1 aggregates, in which the *ρ*(**r**) values at critical points 2 (Figure 13) between the nitrogen atoms of the NO and phthalocyanine molecules are the largest and exceed 0.01 a.u. (Table S1, Supplementary Materials). Although it was noted above that these aggregates are of no interest for the interpretation of experimental data in view of the impossibility of nitric oxide penetration into the stack between phthalocyanines, it is the method of the NO molecule coordination with the bridging nitrogen atom that is of interest. Such bonding is energetically more favorable and, therefore, should have a greater effect on the electrical conductivity of thin films. In this regard, we considered aggregates consisting of two phthalocyanine molecules linked by *π*-*π*-interaction, 2MPcR_4_/NO (Figure 15). In these, the NO molecule is located above two bridging nitrogen atoms and, due to the strong binding of macrocycles, cannot penetrate between them. This model seems to be more correct and suitable for the interpretation of experimental data.

It was found as a result of the calculations that, in a series of 2MPcCl_4_/NO, 2MPc/NO, and 2MPcF_4_/NO aggregates, regardless of the nature of the metal atom, the absolute value of binding energy of the NO molecule with phthalocyanines decreases (Table 3). Moreover, when passing from MPcCl_4_ to MPc, this value changes more significantly (by 0.08 eV) than when passing from MPc to MPcF_4_ (by 0.03–0.04 eV). At the same time, the absolute value of the NO molecule binding energy is slightly higher (by 0.01–0.02 eV) in the case of aggregates with iron phthalocyanines than in the case of those with cobalt phthalocyanines. This is in good agreement with experimental results, which show firstly that the sensitivity of films of chloro-substituted phthalocyanines is much higher than films of MPcR_4_ with other substituents. This slightly differs, although higher, in the case of unsubstituted phthalocyanines compared to fluorine-substituted ones. Secondly, iron phthalocyanines have a stronger sensor response compared to the corresponding cobalt phthalocyanines. However, it is worth noting here that the difference in the NO molecule binding energies in 2FePcR_4_/NO and 2CoPcR_4_/NO, which is 0.01–0.02 eV, is quite small when compared with the difference in the case of different substituents. However, the differences in the sensor response values of iron and cobalt phthalocyanines are more significant. This effect, as noted earlier [28], may be due to the fact that, in addition to the binding energy, when performing quantum-chemical calculations, it is also necessary to consider changes in the electronic structure of films during the gas molecule adsorption. This can be realized in the future in the process of performing calculations of phthalocyanine stacks in the form of periodic systems.

The nitric oxide interaction with phthalocyanine dimers is accompanied by the appearance of six bond critical points (Figure 15), two of which are between two hydrogen atoms and an oxygen atom and four between the nitrogen atom of the NO molecule, two hydrogen atoms and two bridging nitrogen atoms of phthalocyanines. The values of ρ(**r**), ∇^2^ρ(**r**), and h(**r**) are generally similar to those observed in the case of MPcR_4_/NO-m (Table S2, Supplementary Materials), which indicates the binding of NO to dimers by van der Waals forces. In this case, the strongest interaction is described by BCPs 6 (Figure 15) between the nitrogen atom of the NO molecule and the bridging nitrogen atom of one of the two phthalocyanines. The electron density values at these points are in the range of 0.017–0.020 a.u. (Table S2, Supplementary Materials).

It was previously shown [23,30] that NH_3_ interacted with metal phthalocyanines through the formation of hydrogen bonds between the ammonia hydrogen atom and the nitrogen bridge of a phthalocyanine. Here we showed that NO interacted through the formation of van der Waals bonds. Stronger hydrogen bonds may indicate a stronger interaction and a stronger change in the electrical conductivity of thin films during the binding of NH_3_, and, as a consequence, a greater sensor response to ammonia than to nitric oxide, as shown by studies of the sensor response to interfering gases (Figure 12). At the same time, MPcCl_4_ films have less sensor response to CO_2_. Similar to NO, CO_2_ molecule do not have hydrogen atoms and can interact with phthalocyanine molecules only via the formation of van der Waals bonds; however they are not polar. For this reason, the bonds appear to be weaker than in the case of NO.

## 4. Conclusions

In this work, the effect of substituents in cobalt(II) and iron(II) phthalocyanines (CoPcR_4_ and FePcR_4_ with R = H, F, Cl, tBu) on the structural features of their films and their chemi-resistive sensor response to low concentration of nitric oxide were studied. For the correct interpretation of XRD patterns of phthalocyanine films, structures of CoPcCl_4_ and FePcCl_4_ single crystals were determined for the first time. It was shown that CoPcCl_4_ molecules packed with a “herringbone” pattern, which is similar to β-CoPc, but with the packing angle closer to α-CoPc. FePcCl_4_ was isostructural to FePcF_4_, with molecules packed in uniform stacks. Both CoPcCl_4_ and FePcCl_4_ exhibited polymorphism, which was different from their tetra-fluorinated analogs. All studied cobalt(II) and iron(II) phthalocyanines formed oriented polycrystalline films with various degrees of crystallinity when deposited onto glass substrate.

All films were tested as active layers for the determination of a low concentration of nitric oxide. Comparison of the chemi-resistive sensor response of iron and cobalt phthalocyanine films with various substituents showed that the best sensor response was observed for chlorinated derivatives MPcCl_4_ (M = Co, Fe), while the lowest response was for MPc(tBu)_4_ films. FePcCl_4_ films exhibited the maximal response to NO with the detection limit of 3 ppb; the response and recovery times determined at 30 ppb of NO were 30 s and 80 s, respectively. The LOD of a CoPcCl_4_ film was 7 ppb. However, iron phthalocyanine films had lower stability and their sensitivity to NO decreased rapidly over time, while the response of cobalt phthalocyanine films remained stable for at least several months.

In order to explain the obtained regularities, quantum chemical calculations of the binding parameters between NO and phthalocyanine molecules were carried out. It was shown that the binding of NO to the side atoms of phthalocyanines occurred through van der Waals forces, and the values of the binding energies were in direct correlation with the values of the sensor response to NO.

It should be noted that NO is instable in air and easily oxides to NO_2_, which is one of the interfering gases in the process of NO detection. One of the approaches for determining NO in air is the quantitative oxidation of NO to NO_2_, followed by a study of the chemi-resistive sensor response to NO_2_. Thus, further research can be directed to the study of correlations between the response of phthalocyanine films to NO and the response to NO_2_, which is obtained by oxidation of NO.

## Figures and Tables

**Table 1:** Unit cell parameters and refinement statistics for CoPcCl_4_ and FePcCl_4_.

Compound	CoPcCl_4_	FePcCl_4_
Empirical formula	C_32_H_12_Cl_4_CoN_8_	C_32_H_12_Cl_4_FeN_8_
Formula weight	709.23	706.15
Temperature/K	150	150
Crystal system	monoclinic	triclinic
Space group	P2_1_/c	P-1
a/Å	14.0520(16)	3.5971(3)
b/Å	3.6200(4)	13.5180(13)
c/Å	26.611(3)	13.7541(14)
α/°	90	92.487(4)
β/°	94.725(5)	90.116(3)
γ/°	90	97.517(3)
Volume/Å^3^	1349.0(3)	662.41(11)
Z	2	1
ρ_calc_g/cm^3^	1.746	1.770
μ/mm^-1^	1.075	1.017
F(000)	710.0	354.0
Crystal size/mm^3^	0.12 × 0.02 × 0.02	0.04 × 0.02 × 0.005
Radiation	MoKα (λ = 0.71073)	MoKα (λ = 0.71073)
2Θ range for data collection/°	4.4 to 51.37	4.152 to 51.472
Index ranges	−17 ≤ h ≤ 17, 0 ≤ k ≤ 4, 0 ≤ l ≤ 32	−4 ≤ h ≤ 4, −16 ≤ k ≤ 16, −16 ≤ l ≤ 16
Reflections collected	11220	7812
Independent reflections	2932 (R_int_ = 0.0636, R_sigma_ = 0.0640)	2537 (R_int_ = 0.1003, R_sigma_ = 0.1331)
Data/restraints/parameters	2932/0/226	2537/0/225
Goodness-of-fit on F^2^	1.030	0.951
Final R indexes (I >= 2σ (I))	R_1_ = 0.0511, wR_2_ = 0.0983	R_1_ = 0.0571, wR_2_ = 0.0984
Final R indexes (all data)	R_1_ = 0.1023, wR_2_ = 0.1168	R_1_ = 0.1565, wR_2_ = 0.1267
Largest diff. peak/hole/e Å^−3^	0.26/−0.31	0.26/−0.27
CCDC deposition №	2231583	2231584

**Table 2:** Sensor characteristics of MPcR_4_ (M = Co, Fe; R = H, F, Cl, tBu) films to 30 ppb of NO.

Sensing Layer	Sensor Response to 30 ppb of NO	Response/Recovery Time, s	Calculated LOD, ppb
CoPcCl_4_	0.0025	15/120	7
CoPcF_4_	0.0019	20/140	8.5
CoPc	0.0016	40/180	9
CoPc(tBu)_4_	0.0015	20/90	9
FePcCl_4_	0.076	30/80	3
FePcF_4_	0.0065	15/290	5
FePc	0.0087	45/265	4
FePc(tBu)_4_	0.0008	15/105	10

**Table 3:** The NO molecule *E_b_* values (eV) in MPcR_4_/NO-*m* and 2MPcR_4_/NO aggregates.

Aggregate	M = Co				M = Fe			
	m = 1	m = 2	m = 3	m = 4	m = 1	m = 2	m = 3	m = 4
MPc/NO-m	−0.098	−0.025	−0.022	–	−0.102	−0.025	−0.022	–
MPcF4/NO-m	−0.099	−0.028	−0.019	−0.020	−0.101	−0.029	−0.016	−0.021
MPcCl4/NO-m	−0.099	−0.030	−0.042	−0.045	−0.101	−0.030	−0.043	−0.046
2MPc/NO	−0.093				−0.095			
2MPcF4/NO	−0.090				−0.091			
2MPcCl4/NO	−0.101				−0.103			

## Data Availability

Not applicable.

## Supplementary Materials

The following supporting information can be downloaded at: https://www.mdpi.com/article/10.3390/bios13040484/s1, Figure S1. Raman spectra of CoPcCl4 (a) and FePcCl4 (b) films and powders. Differences in the ratio of intensities of some bands may be due to preferential orientation of thin films relative to the substrate surface; Figure S2. 2D AFM images of FePc (a), FePcF4 (b), FePcCl4 (c), and FePc(tBu)4 (d) films; Figure S3. SEM images of FePc (a), FePcF4 (b), FePcCl4 (c), and FePc(tBu)4 (d) films; Table S1: Topological parameters at bond critical points and binding energy of the NO molecule with cobalt and iron phthalocyanines; Table S2: Topological parameters at bond critical points and binding energy of NO molecule with cobalt and iron phthalocyanine dimers.
