# Supplementary material for: Cobalt and Iron Phthalocyanine Derivatives: Effect of Substituents on the Structure of Thin Films and Their Sensor Response to Nitric Oxide
Source: Biosensors (Basel). 2023 Apr 17;13(4):484. doi: 10.3390/bios13040484 (PMC10136685; doi:10.3390/bios13040484)
Supplement: Supplementary file 1 [file biosensors-13-00484-s001.zip › biosensors-2285136-supplementary.pdf]

Cobalt and Iron Phthalocyanine Derivatives: Effect of Substituents on the Structure of Thin Films and Their Sensor Response to Nitric Oxide

Darya Klyamer [1], Wenping Shao [2], Pavel Krasnov [3], Aleksandr Sukhikh [1], Svetlana Dorovskikh [1], Pavel Popovetskiy [1], Xianchun Li [2] and Tamara Basova [1,*]

[1] Nikolaev Institute of Inorganic Chemistry SB RAS, Novosibirsk 630090, Russia; klyamer@niic.nsc.ru (D.K.); a_sukhikh@niic.nsc.ru (A.S.); reter16@yandex.ru (S.D.); popovetskiy@niic.nsc.ru (P.P.)

[2] School of Chemical Engineering, University of Science and Technology Liaoning, Anshan 114051, China; shaowp0318@163.com (W.S.); xianchunli@ustl.edu.cn (X.L.)

[3] International Research Center of Spectroscopy and Quantum Chemistry, Siberian Federal University,
Krasnoyarsk 660074, Russia; kpo1980@gmail.com

* Correspondence: basova@niic.nsc.ru

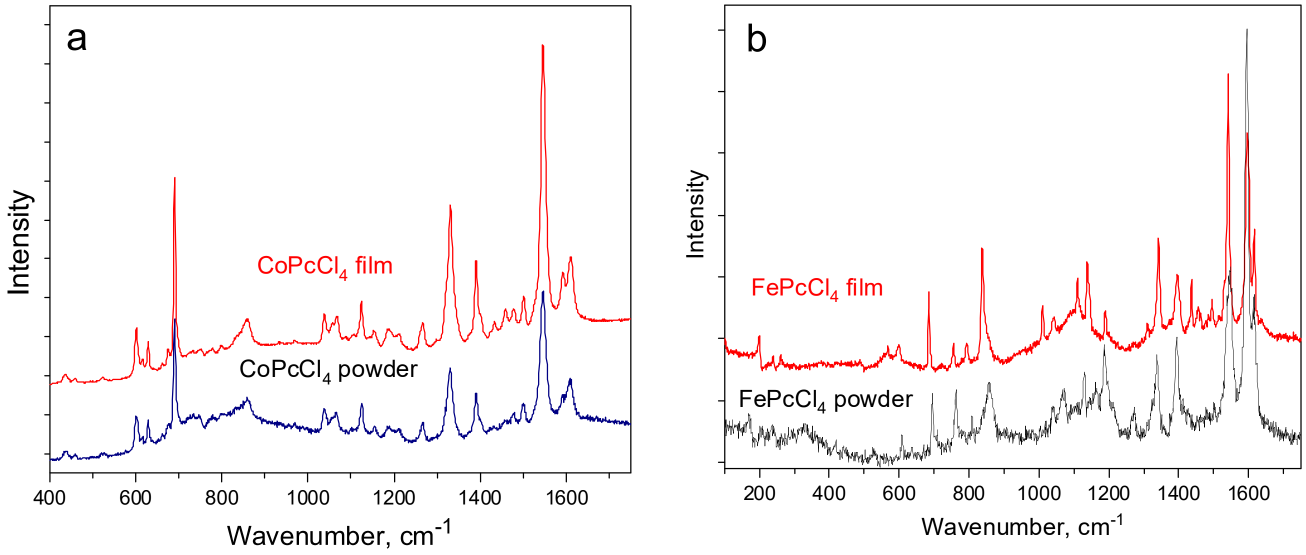

Figure S1. Raman spectra of $CoPcCl_4$ (a) and $FePcCl_4$ (b) films and powders. Differences in the ratio of intensities of some bands may be due to preferential orientation of thin films relative to the substrate surface.

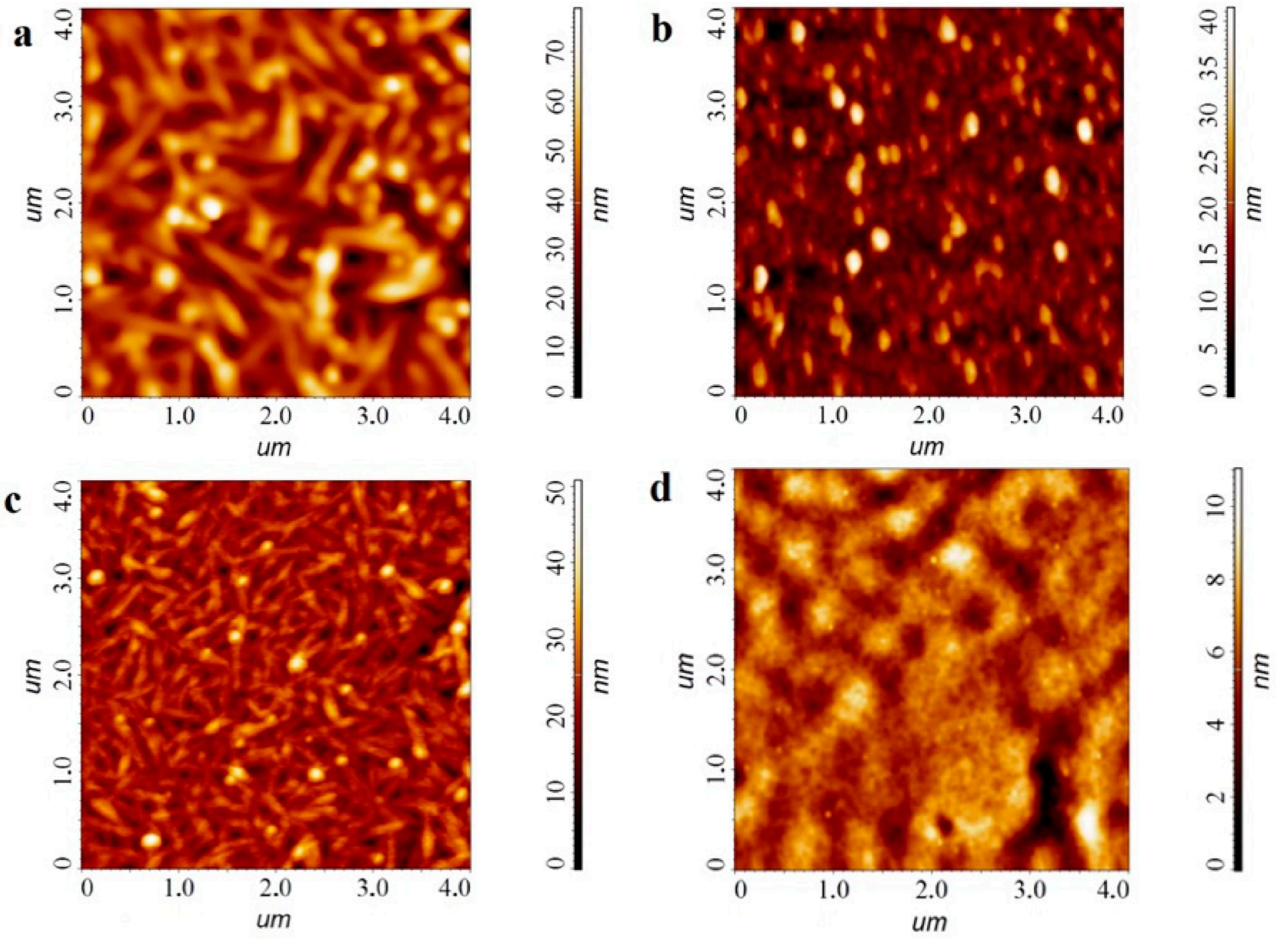

Figure S2. 2D AFM images of FePc (a), FePcF$_4$ (b), FePcCl$_4$ (c), and FePc(tBu)$_4$ (d) films.

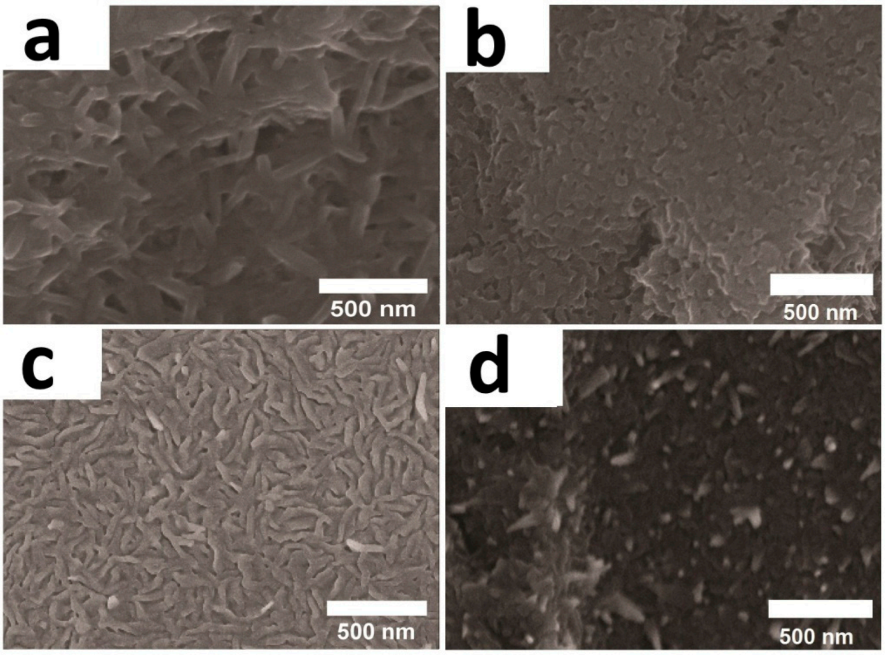

Figure S3. SEM images of FePc (a), FePcF$_4$ (b), FePcCl$_4$ (c), and FePc(tBu)$_4$ (d) films.

Table S1. Topological parameters at bond critical points and binding energy of the NO molecule with cobalt and iron phthalocyanines.

Aggregate	BCP	M = Co			M = Fe		
		$\rho(\mathbf{r})$, a.u.	$\nabla^2\rho(\mathbf{r})$, a.u.	$h(\mathbf{r})$, a.u.	$\rho(\mathbf{r})$, a.u.	$\nabla^2\rho(\mathbf{r})$, a.u.	$h(\mathbf{r})$, a.u.
MPc/NO-1	1	$7.1\cdot10^{-3}$	$2.3\cdot10^{-2}$	$1.3\cdot10^{-3}$	$7.3\cdot10^{-3}$	$2.4\cdot10^{-2}$	$1.3\cdot10^{-3}$
	2	$1.5\cdot10^{-2}$	$5.4\cdot10^{-2}$	$2.6\cdot10^{-3}$	$1.6\cdot10^{-2}$	$5.5\cdot10^{-2}$	$2.6\cdot10^{-3}$
	3	$7.0\cdot10^{-3}$	$2.3\cdot10^{-2}$	$1.3\cdot10^{-3}$	$7.4\cdot10^{-3}$	$2.4\cdot10^{-2}$	$1.3\cdot10^{-3}$
MPc/NO-2	1	$6.3\cdot10^{-3}$	$1.9\cdot10^{-2}$	$9.3\cdot10^{-4}$	$6.4\cdot10^{-3}$	$1.9\cdot10^{-2}$	$9.4\cdot10^{-4}$
	2	$5.7\cdot10^{-3}$	$2.0\cdot10^{-2}$	$9.8\cdot10^{-4}$	$5.8\cdot10^{-3}$	$2.0\cdot10^{-2}$	$9.9\cdot10^{-4}$
MPc/NO-3	1	$6.2\cdot10^{-3}$	$1.9\cdot10^{-2}$	$9.1\cdot10^{-4}$	$6.6\cdot10^{-3}$	$2.0\cdot10^{-2}$	$9.6\cdot10^{-4}$
	2	$5.2\cdot10^{-3}$	$1.8\cdot10^{-2}$	$8.7\cdot10^{-4}$	$5.2\cdot10^{-3}$	$1.8\cdot10^{-2}$	$8.7\cdot10^{-4}$
$MPcF_4$/NO-1	1	$7.0\cdot10^{-3}$	$2.3\cdot10^{-2}$	$1.3\cdot10^{-3}$	$7.3\cdot10^{-3}$	$2.4\cdot10^{-2}$	$1.3\cdot10^{-3}$
	2	$1.5\cdot10^{-2}$	$5.3\cdot10^{-2}$	$2.6\cdot10^{-3}$	$1.5\cdot10^{-2}$	$5.4\cdot10^{-2}$	$2.6\cdot10^{-3}$
	3	$7.3\cdot10^{-3}$	$2.4\cdot10^{-2}$	$1.3\cdot10^{-3}$	$7.4\cdot10^{-3}$	$2.4\cdot10^{-2}$	$1.3\cdot10^{-3}$
$MPcF_4$/NO-2	1	$6.5\cdot10^{-3}$	$2.0\cdot10^{-2}$	$9.7\cdot10^{-4}$	$6.3\cdot10^{-3}$	$1.9\cdot10^{-2}$	$9.3\cdot10^{-4}$
	2	$6.1\cdot10^{-3}$	$2.1\cdot10^{-2}$	$1.1\cdot10^{-3}$	$5.8\cdot10^{-3}$	$2.0\cdot10^{-2}$	$9.9\cdot10^{-4}$
$MPcF_4$/NO-3	1	$6.8\cdot10^{-3}$	$2.2\cdot10^{-2}$	$1.2\cdot10^{-3}$	$6.9\cdot10^{-3}$	$2.2\cdot10^{-2}$	$1.2\cdot10^{-3}$
	2	$7.0\cdot10^{-3}$	$2.9\cdot10^{-2}$	$1.5\cdot10^{-3}$	$6.8\cdot10^{-3}$	$2.9\cdot10^{-2}$	$1.5\cdot10^{-3}$
$MPcF_4$/NO-4	1	$6.4\cdot10^{-3}$	$2.1\cdot10^{-2}$	$1.1\cdot10^{-3}$	$6.8\cdot10^{-3}$	$2.2\cdot10^{-2}$	$1.2\cdot10^{-3}$
	2	$7.3\cdot10^{-3}$	$3.1\cdot10^{-2}$	$1.6\cdot10^{-3}$	$7.0\cdot10^{-3}$	$3.0\cdot10^{-2}$	$1.6\cdot10^{-3}$
$MPcCl_4$/NO-1	1	$7.7\cdot10^{-3}$	$2.5\cdot10^{-2}$	$1.4\cdot10^{-3}$	$7.7\cdot10^{-3}$	$2.5\cdot10^{-2}$	$1.4\cdot10^{-3}$
	2	$1.5\cdot10^{-2}$	$5.1\cdot10^{-2}$	$2.6\cdot10^{-3}$	$1.5\cdot10^{-2}$	$5.2\cdot10^{-2}$	$2.6\cdot10^{-3}$
	3	$8.0\cdot10^{-3}$	$2.6\cdot10^{-2}$	$1.4\cdot10^{-3}$	$7.9\cdot10^{-3}$	$2.6\cdot10^{-2}$	$1.4\cdot10^{-3}$
$MPcCl_4$/NO-2	1	$6.4\cdot10^{-3}$	$1.9\cdot10^{-2}$	$9.6\cdot10^{-4}$	$6.6\cdot10^{-3}$	$2.0\cdot10^{-2}$	$9.9\cdot10^{-4}$
	2	$6.0\cdot10^{-3}$	$2.1\cdot10^{-2}$	$1.1\cdot10^{-3}$	$6.0\cdot10^{-3}$	$2.1\cdot10^{-2}$	$1.0\cdot10^{-3}$
$MPcCl_4$/NO-3	1	$7.9\cdot10^{-3}$	$2.5\cdot10^{-2}$	$1.3\cdot10^{-3}$	$7.8\cdot10^{-3}$	$2.5\cdot10^{-2}$	$1.3\cdot10^{-3}$
	2	$9.0\cdot10^{-3}$	$3.3\cdot10^{-2}$	$1.9\cdot10^{-3}$	$8.7\cdot10^{-3}$	$3.2\cdot10^{-2}$	$1.9\cdot10^{-3}$
$MPcCl_4$/NO-4	1	$8.2\cdot10^{-3}$	$2.6\cdot10^{-2}$	$1.4\cdot10^{-3}$	$8.5\cdot10^{-3}$	$2.7\cdot10^{-2}$	$1.4\cdot10^{-3}$
	2	$9.3\cdot10^{-3}$	$3.4\cdot10^{-2}$	$2.0\cdot10^{-3}$	$8.3\cdot10^{-3}$	$3.0\cdot10^{-2}$	$1.8\cdot10^{-3}$

Table S2. Topological parameters at bond critical points and binding energy of NO molecule with cobalt and iron phthalocyanine dimmers.

Aggregate	BCP	M = Co			M = Fe		
		$\rho(\mathbf{r})$, a.u.	$\nabla^2\rho(\mathbf{r})$, a.u.	$h(\mathbf{r})$, a.u.	$\rho(\mathbf{r})$, a.u.	$\nabla^2\rho(\mathbf{r})$, a.u.	$h(\mathbf{r})$, a.u.
2MPc/NO	1	$7.6\cdot10^{-3}$	$2.9\cdot10^{-2}$	$1.6\cdot10^{-3}$	$7.8\cdot10^{-3}$	$3.0\cdot10^{-2}$	$1.6\cdot10^{-3}$
	2	$7.3\cdot10^{-3}$	$3.0\cdot10^{-2}$	$1.7\cdot10^{-3}$	$7.7\cdot10^{-3}$	$3.2\cdot10^{-2}$	$1.8\cdot10^{-3}$
	3	$9.1\cdot10^{-3}$	$2.9\cdot10^{-2}$	$1.5\cdot10^{-3}$	$9.5\cdot10^{-3}$	$3.1\cdot10^{-2}$	$1.6\cdot10^{-3}$
	4	$4.6\cdot10^{-3}$	$1.4\cdot10^{-2}$	$7.8\cdot10^{-4}$	$4.3\cdot10^{-3}$	$1.3\cdot10^{-2}$	$7.3\cdot10^{-4}$
	5	$3.4\cdot10^{-3}$	$1.2\cdot10^{-2}$	$7.5\cdot10^{-4}$	$3.7\cdot10^{-3}$	$1.3\cdot10^{-2}$	$8.2\cdot10^{-4}$
	6	$1.9\cdot10^{-2}$	$6.5\cdot10^{-2}$	$2.7\cdot10^{-3}$	$2.0\cdot10^{-2}$	$6.7\cdot10^{-2}$	$2.7\cdot10^{-3}$
2MPcF$_4$/NO	1	$7.2\cdot10^{-3}$	$2.7\cdot10^{-2}$	$1.5\cdot10^{-3}$	$7.5\cdot10^{-3}$	$2.8\cdot10^{-2}$	$1.6\cdot10^{-3}$
	2	$7.4\cdot10^{-3}$	$3.1\cdot10^{-2}$	$1.8\cdot10^{-3}$	$7.8\cdot10^{-3}$	$3.3\cdot10^{-2}$	$1.8\cdot10^{-3}$
	3	$9.6\cdot10^{-3}$	$3.1\cdot10^{-2}$	$1.6\cdot10^{-3}$	$1.0\cdot10^{-2}$	$3.2\cdot10^{-2}$	$1.6\cdot10^{-3}$
	4	$4.8\cdot10^{-3}$	$1.5\cdot10^{-2}$	$8.3\cdot10^{-4}$	$4.4\cdot10^{-3}$	$1.4\cdot10^{-2}$	$7.5\cdot10^{-4}$
	5	$3.5\cdot10^{-3}$	$1.2\cdot10^{-2}$	$7.6\cdot10^{-4}$	$3.7\cdot10^{-3}$	$1.3\cdot10^{-2}$	$8.1\cdot10^{-4}$
	6	$1.7\cdot10^{-2}$	$5.9\cdot10^{-2}$	$2.7\cdot10^{-3}$	$1.8\cdot10^{-2}$	$6.1\cdot10^{-2}$	$2.7\cdot10^{-3}$
2MPcCl$_4$/NO	1	$6.9\cdot10^{-3}$	$2.6\cdot10^{-2}$	$1.4\cdot10^{-3}$	$7.2\cdot10^{-3}$	$2.8\cdot10^{-2}$	$1.5\cdot10^{-3}$
	2	$7.3\cdot10^{-3}$	$3.1\cdot10^{-2}$	$1.8\cdot10^{-3}$	$7.6\cdot10^{-3}$	$3.2\cdot10^{-2}$	$1.8\cdot10^{-3}$
	3	$9.5\cdot10^{-3}$	$3.0\cdot10^{-2}$	$1.5\cdot10^{-3}$	$9.8\cdot10^{-3}$	$3.1\cdot10^{-2}$	$1.6\cdot10^{-3}$
	4	$4.9\cdot10^{-3}$	$1.5\cdot10^{-2}$	$8.5\cdot10^{-4}$	$4.6\cdot10^{-3}$	$1.4\cdot10^{-2}$	$8.0\cdot10^{-4}$
	5	$3.5\cdot10^{-3}$	$1.2\cdot10^{-2}$	$7.6\cdot10^{-4}$	$3.7\cdot10^{-3}$	$1.3\cdot10^{-2}$	$8.1\cdot10^{-4}$
	6	$1.7\cdot10^{-2}$	$5.9\cdot10^{-2}$	$2.7\cdot10^{-3}$	$1.7\cdot10^{-2}$	$5.9\cdot10^{-2}$	$2.7\cdot10^{-3}$
